# Protective role of 1,25(OH)_2_vitamin D_3_ in the mucosal injury and epithelial barrier disruption in DSS-induced acute colitis in mice

**DOI:** 10.1186/1471-230X-12-57

**Published:** 2012-05-30

**Authors:** Hongwei Zhao, Hong Zhang, Hui Wu, Hui Li, Lei Liu, Jian Guo, Chenyang Li, David Q Shih, Xiaolan Zhang

**Affiliations:** 1 Department of Gastroenterology, The Second Hospital of Hebei Medical University, Hebei Key Laboratory of Gastroenterology, Hebei Institute of Gastroenterology, No.215 Heping West Road, 050000, Shijiazhuang, China; 2 Hebei Medical University, No.361 Zhongshan East Road, 050017, Shijiazhuang, China; 3 Inflammatory Bowel & Immunobiology Research Institute, Cedars-Sinai Medical Center, Los Angeles, CA, 90048, USA

**Keywords:** Barrier protection function, Dextran sulfate sodium, Inflammatory bowel disease, Tight junction, 1,25-dihydroxyvitamin D_3_

## Abstract

**Background:**

Intestinal hyper-permeability plays a critical role in the etiopathogenesis of inflammatory bowel disease (IBD) by affecting the penetration of pathogens, toxic compounds and macromolecules. 1,25-dihydroxyvitamin D_3_ [1,25(OH)_2_D_3_], the active form of vitamin D, has been shown to be an important regulator of IBD and recent epidemiology suggests that patients with IBD have an impaired vitamin D status. The purpose of this study is to investigate the possible protective effects of 1,25(OH)_2_D_3_ on mucosal injury and epithelial barrier disruption on dextran sulfate sodium (DSS)-induced acute colitis model.

**Methods:**

We used DSS-induced acute colitis model to investigate the protective effects of 1,25(OH)_2_D_3_ on mucosal injury and epithelial barrier integrity. Severity of colitis was evaluated by disease activity index (DAI), body weight (BW) change, colon length, histology, myeloperoxidase (MPO) activity, and proinflammatory cytokine production including tumor necrosis factor-α (TNF-α) and interferon-γ (IFN-γ). *In vitro* the protective role of 1,25(OH)_2_D_3_ was assessed by incubating Caco-2 cells with or without DSS and measuring transepithelial electrical resistance (TEER) and fluorescein isothiocyanate dextran (FITC-D). The intestinal permeability was analyzed by FITC-D, bacterial translocation and measurement of lipopolysaccharide (LPS). Ultrastructural features of the colon tissue and Caco-2 cell monolayer were observed by electron microscopy. Expressions of tight junction (TJ) proteins in the colon mucosa and Caco-2 cells were detected by immunohistochemistry, immunofluorescence, Western blot and real-time fluorescent quantitative PCR, respectively.

**Results:**

DSS-induced acute colitis model was characterized by a reduced BW, AUC of BW, serum calcium, higher DAI, AUC of DAI, shortened colon length, elevated MPO activity, worsened histologic inflammation, increased mononuclear cell numbers in mesenteric lymph nodes (MLNs) and colonic lamina propria (LP), and enhanced proteins and mRNA levels of TNF-α and IFN-γ. 1,25(OH)_2_D_3_ markedly increased expressions of TJ proteins and mRNA and decreased the FITC-D permeability and the level of LPS. Furthermore, 1,25(OH)_2_D_3_ abrogated bacterial translocation to MLNs and ameliorated ultrastructural features of the colon epithelium by scanning electron microscopy (SEM). *In vitro*, 1,25(OH)_2_D_3_ increased TEER, TJ proteins and mRNA expressions, decreased the FITC-D permeability, and preserved structural integrity of the TJ in Caco-2 cells.

**Conclusions:**

1,25(OH)_2_D_3_ may play a protective role in mucosal barrier homeostasis by maintaining the integrity of junction complexes and in healing capacity of the colon epithelium. 1,25(OH)_2_D_3_ may represent an attractive and novel therapeutic agent for the adjuvant therapy of IBD.

## Background

Inflammatory bowel disease (IBD), comprised of Crohn’s disease (CD) and ulcerative colitis (UC), are chronic diseases characterized by aberrant immune responses to luminal bacteria in genetically susceptible subjects [1]. Although the exact pathogenesis of IBD is unknown, the initiation of inflammation and relapse of disease activity are associated with engagement of the innate and adpative immune responses, including increased production in tumor necrosis factor-α (TNF-α) and interferon-γ (IFN-γ) in the intestine [2,3]. These pro-inflammatory cytokines are potential pathogenic factors, which impair mucosal barrier function and intestinal permeability. A disproportionate large number of first-degree relatives of patients with IBD have increased intestinal permeability, which suggests barrier dysfunction may be an early defect. Previous studies have demonstrated a decreased expression of junction complex proteins in the intestinal mucosa of patients with IBD [4,5]. Impaired gut epithelial barrier function may lead to persistent immune reactions, thus augmenting the gut inflammation [6].

The intestinal epithelial barrier consists of epithelial cells and intercellular junctions [7-9]. Tight junction (TJ) is the apical-most intercellular structure in epithelial cells, accounting for the cell-cell adhesion, polarity, and permeability barrier to paracellular transport of the solutes [10]. The TJ creates a semipermeable barrier, separating different organ compartments. It is composed of a large number of protein components, such as occludins, claudins and zonula occludens-1 (zo-1). Both claudins and occludins are coupled either directly or indirectly to cytoskeleton actin filaments with zo-1 and other factors. Distributions of claudins vary along the gut epithelial cells and express different types in the connecting cells through the formation of zipper structures [11,12]. Once the mucosal barrier is breached, the submucosa is exposed to a vast pool of luminal antigens, including food and bacteria, and the innate immune responses are engaged to produce large amounts of cytokines. Therefore, maintaining TJ and barrier function may provide potential benefits in the adjuvant therapy of many gastrointestinal diseases, including IBD [13-15].

1,25-dihydroxyvitamin D_3_ [1,25(OH)_2_D_3_, the active form of vitamin D, has been shown to be an important regulator in many experimental autoimmune diseases [16-19]. A recent study has shown that vitamin D directly modulates the T-cell antigen receptor (TCR) [20]. In naive T cells, low expression of phospholipase C (PLC)-γ1 is correlated with low TCR responsiveness. The induction of PLC-γ1 was shown to be dependent on vitamin D and vitamin D receptor (VDR) [21]. Studies have implicated that the deficiency of VDR may lead to exaggerated inflammatory responses [22,23]. Consistent with its anti-inflammatory role, 1,25(OH)_2_D_3_ down-regulates the expressions of many pro-inflammatory cytokines, such as TNF-α, IFN-γ, and other cytokines [24,25]. 1,25(OH)_2_D_3_ is also important to maintain homeostasis in the respiratory tract, skin, and blood–brain and blood-retinal barrier development. 1,25(OH)_2_D_3_ has been found to protect the intestinal mucosa from various insults, combination therapy with angiotensin II type 1 (AT II -1). Vitamin D analog markedly restored glomerular filtration barrier structure, and the combined treatment reversed the decline of slit diaphragm proteins, such as nephrin and zo-1. Hence, 1,25(OH)_2_D_3_ may prevent and treat these conditions [26-28].

The role of 1,25(OH)_2_D_3_ in the regulation of intestinal barrier integrity and innate immune response in DSS-induced acute colitis is unclear. Herein we hypothesized that 1,25(OH)_2_D_3_ may play an important role in maintaining the integrity of the intestinal mucosal barrier and regulate the innate immunity, which implicates vitamin D metabolic pathway is a novel target for the adjuvant therapy of IBD.

## Methods

### Ethics statement

The mice and the protocol involved in the study had been approved by Institutional Animal Care and Use Committee (IACUC). Approval ID: I07-038-3. All the mice were housed under standard conditions per protocols of IACUC and Hebei medical university vivarium in a barrier facility (GB 14925-2001).

### Animal studies

C57BL/6 mice were purchased from Vital River Laboratory Animal Technology Co. Ltd for *in vivo* studies [License No. SCXK (Beijing) 2006-0009]. Acute colitis was induced by administration of dextran sodium sulfate (DSS; 40 000-50 000 MW; Sigma) drinking water. Male mice of 8 weeks received either regular drinking water (control) or 2% (w/v) DSS drinking water (model) ad libitum for 7 days, after which the mice were resumed on water for the remainder of the experiment. A total of 30 mice were randomly assigned to control, model and 1,25(OH)_2_D_3−_treated group (each group = 10). The mice in the 1,25(OH)_2_D_3−_treated group received 1,25(OH)_2_D_3_ (Sigma) daily (0.2 ug/25 g/d) by intragastric administration for 14 days [29], and the mice in control and model group were given normal saline without DSS.

### Serum measurements

Mice were executed at the end of the experiment. Serum was obtained, and Lipopolysaccharide (LPS) kit was purchased from Sigma, and Calcium (587-A) kit was purchased from Sigma. Serum calcium was measured according to the manufacturer’s instructions. Vitamin D deficiency was monitored by serum calcium analysis and normal serum calcium levels for mice are 2.00–2.75 mmol/L [29]. LPS was measured by Limulus quantitative azo color (LQAC) test.

### Assessment of disease activity

Rachmilewitz DAI was assessed by an investigator blinded to the protocol according to a standard scoring system. The combined score composes of the extent of body weight (BW) loss, stool consistency and detection of occult blood (OB) in the stool, and they are defined as follows. Loss in BW is scored as: no weight loss is scored as 0, weight loss of 1–5% from baseline as 1, 5–10% as 2, 10–20% as 3, and more than 20% as 4. For stool consistency, a score of 0 is assigned for wellformed pellets, 2 points for pasty and semiformed stools that do not adhere to the anus, and 4 points for liquid stools that were adhere to the anus. For OB, a score of 0 point is assigned for no blood, 2 points for positive hemoccult, and 4 points for gross bleeding. These scores are added together and divided by three, resulting in DAI ranging from 0 (healthy) to 4 (maximal activity of colitis) [30] and measured by area under curve (AUC).

### Assessment of colonic injury and inflammation

Postmortem, the colon from the cecum to the anus was removed, and the entire colon length was measured as a marker of inflammation. On macroscopic examination, the degree of colon damage was scored as follows: 0, normal colon tissue; 1, minimal colon wall thickening without congestion; 2, moderate colon wall thickening with congestion; 3, moderate colon wall thickening, rigidity, and congestion; and 4, marked colon wall thickening, rigidity, and congestion. A segment of the proximal colon was fixed in 10% formalin, and embedded in paraffin; 4 um-thick sections of this tissue were stained with hematoxylin and eosin (H&E). Histologic evaluation was performed by two investigators blinded to the animal groups and the inflammation was graded as follows: severity of inflammation (0–3: none, slight, moderate, severe), extent of injury (0–3: none, mucosal, mucosal and submucosal, transmural), and crypt damage (0–4: none, basal 1/3 damaged, basal 2/3 damaged, only surface epithelium intact, entire crypt and epithelium lost). Then each score was multiplied by a equivalent with the percentage of tissue involved (×1: 0–25%, ×2: 26–50%, ×3: 51–75%, ×4: 76–100%).

### Determination of myeloperoxidase (MPO) activity

MPO activity in homogenates of the colon was determined in this way: equal weights (100 mg wet weight) of the colon from each group were suspended in 1 mL buffer (0.5% hexadecyltrimethylammonium bromide in 50 mM phosphate buffer, pH 6.0) and sonicated at 30 cycles, twice for 30 s on ice. Homogenates were centrifuged, 2000 g, 4°C, and the supernatants were stored at 80°C. The samples were incubated with a substrate of odianisidine hydrochloride and the reaction was carried out in a 96-well plate by adding to 290 μL 50 mM phosphate buffer, 3 μL substrate solution (containing 20 mg/mL odianisidine hydrochloride), and 3 μL H_2_O_2_ (20 mM). The samples (10 μL each well) were added to each well to start the reaction. The reaction was stopped by adding 3 μL sodium azide (30%), and the plates were read for the assay at light absorbance of 460 nm. MPO activity was determined by the curve obtained from the standard MPO [31].

### Isolation of mesenteric lymph nodes cells (MLNs) and lamina propria mononuclear cells (LPMCs)

Mesenteric lymph nodes (MLNs) were placed in a petri dish with 2 mL ice-cold RPMI 1640. Two prewet sterile glass slides were used to smash MLNs, and then passed through a 70 μm nylon cell strainer into a 50 mL Falcon tube. Petri dish and glass slides were rinsed with an additional 3 mL RPMI 1640, centrifuged, removed the supernatant, and resuspended the pellet in 1.0 mL RPMI 1640 with 10% FCS. The cells were then counted. Isolation of lamina propria mononuclear cells (LPMCs) was described as follows: The dissected gut was opened longitudinally, transferred onto HBSS/5 mM EDTA and incubated for 20 min at 37°C under slow rotation (40 g) in a thermal incubator. Gut tissues were then transferred onto a new petri dish with 1–2 mL collagenase solution (100 mL of RPMI 1640 with 10% FCS [32]: Hyaluronidase, 0.25 g; Collagenase type II, 0.15 g; DNase I, 0.025 g) and minced into about 1 mm-square pieces. The minced tissues were then passed through a 70 um cell strainer, then centrifuged, resuspended the pellet in 6 ml of 45% Percoll solution, overlaid the cell suspension on top of 3 mL of the 72% Percoll solution, centrifuged, carefully collected the cells, centrifuged again, aspirated and resuspended the cells immediately in 500 μL RPMI 1640 with 10% FCS, and at last counted cells one by one [33].

### Immunohistochemistry/immunofluorescence

For immunohistochemistry staining, antigens were retrieved by 10 min boiling in 10 mM citrate (pH 6.0). The slides were stained with mouse monoclonal anti-claudin-1 antibody, anti-occludin antibody (Santa Cruez), and rabbit polyclonal anti-zo-1 antibody (Invitrogen), rabbit polyclonal anti-IFN-γ antibody (Santa Cruez) and mouse monoclonal anti-TNF-α antibody (sigma). After incubation with peroxidase-conjugated secondary antibody, signals were visualized with a diaminobenzidine (DAB) peroxidase substrate kit (Vector Laboratories). For TJ immunofluorescence staining, Caco-2 cell monolayer (fixed in 95% ethanol) or colon sections were incubated with mouse monoclonal anti-claudin-1 antibody, anti-occludin antibody (Santa Cruez) and rabbit polyclonal anti-zo-1 antibodies (Invitrogen), then with an FITC-conjugated secondary antibody or Cy3-conjugated secondary antibody. Slides were examined with a Leica DMIRE2 scanning laser confocal microscope.

### *In vivo* permeability

*In vivo* permeability assay was performed to assess barrier function and performed by fluorescein isothiocyanate dextran (FITC-D). Briefly, food and water were withdrawn for 4 h, and mice were gavaged with permeability tracer (60 mg/100 g BW of FITC-D (MW 4 000; Sigma). Serum was collected retroorbitally 4 h after FITC-D gavaged, and fluorescence intensity was measured (excitation, 492 nm; emission, 525 nm; Cytofluor 2 300 nm; Millipore), and FITC-D concentrations were determined from standard curves generated by serial dilution of FITC-D [34]. Permeability was calculated by linear regression of sample fluorescence (Excel 5.0, Microsoft Office). Detection of viable bacteria in MLNs represented bacterial translocation from the lumen to the MLNs. The MLNs of left colonic regions were removed aseptically and dissected free of fat. A 0.1 ml aliquot of each homogenate was plated into blood agar, incubated at 37°C for 48 h, and then counted the number of colonies. The ratio of bacterial translocation was presented for percentage.

### Electron microscopy

Specimens were fixed, washed in acetone, critical point dried, coated with gold by a sputter coater, and observed under scanning electron microscope (SEM) fitted with a lanthanum hexaboride cathode using an accelerating voltage of 10 kV. Cell monolayer samples were fixed in 2% glutaraldehyde and postfixed in 1% osmium tetroxide in 0.1 M phosphate buffer, pH 7.4, 1 h, 37°C. The tissues were dehydrated in an ascending series of ethanol, infiltrated with eponate 12 resin and then embedded and polymerized, 70°C, 24 h. Resin-embedded blocks were sectioned at 70 nm and collected on 200 mesh, formvar-coated copper grids. Grids teue were stained with uranyl acetate and lead citrate and examined with a JEOL 1200 EX II transmission electron microscope (TEM).

### Caco-2 cell culture

The Human Colon Carcinoma cell Line (Caco-2) was purchased from Shanghai Institutes of Biochemistry and Cell Biology for *in vitro* studies, and cultured in medium supplemented with 10% fetal bovine serum (FBS) and penicillin (50 U/ml)/streptomycin (50 μg/mL) in a 5% CO_2_ atmosphere at 37°C. Caco-2 cell monolayer was incubated with or without 2% DSS in the absence or presence of 10^−9^, 10^−8^, 10^−7^ M of 1,25(OH)_2_D_3_ (administered 2 h prior to DSS) for 48 h.

### *In vitro* permeability

Paracellular permeability was determined by measuring the apical to the basolateral flux of FITC-D using a modification of previously described method [34]. Briefly, confluent epithelial monolayer on a 0.33 cm^2^, 0.4 μm pore size permeable support was washed twice with Hanks’ balanced salt solution containing calcium chloride and magnesium sulfate (HBSS) and maintained at 37°C on a shaking warm plate. FITC-D, 1 mg/mL, was added apically at time 0, and 50 μL samples were removed from the basolateral compartment at 30 min intervals from 0–360 min, inclusively. Fluorescence intensity of each sample was measured (excitation, 492 nm; emission, 525 nm; Cytofluor 2 300 nm; Millipore Corp, Waters Chromatography, Bedford, MA), and FITC-D concentrations were determined from standard curves generated by serial dilution of FITC-D. Paracellular flux was calculated by linear regression of sample fluorescence (Excel 5.0, Microsoft WA, Power Macintosh 7200).

### TEER measurement

For transepithelial electric resistance (TEER) measurement, Caco-2 cells were cultured on collagen-coated transwell polycarbonate membrane filter inserts (Corning). The cells were seeded at a density of 1 × 10^5^ cell/mL, the medium was changed every 1 or 2 days. The integrity of the monolayer was observed to accomplish after culturing for 21 days, and evaluated by measuring TEER with Millicell-Electrical Resistance System (ERS) equipment (Millipore). Monolayer showing TEER values of 130–200 ohm/cm^2^ were used for the experiments. The monolayer cells were gently rinsed three times with HBSS and equilibrated in the same solution for 30 min at 37°C (inside volume; 400 μL, outside volume; 600 μL). An aliquot (40 μL) of the apical solution was replaced by the same volume of sample solution, containing each compound at concentrations of 100–800 μg/mL, and the TEER value of the monolayer was monitored for up to 360 min after adding to the sample solution [35].

### Real-time fluorescent quantitation PCR (real time Q-PCR)

The expressions of the gene zo-1, occludin, claudin-1, TNF-α and IFN-γ were characterized by real time Q-PCR. Briefly, Total RNA was extracted from colons or Caco-2 cells using Trizol (Gibco) reversely transcripted into cDNA according to manufacture’s protocol. The primers for the zo-1, occludin and claudin-1 genes were as follows: zo-1-Forward 5′-TCATCCCAAATAAGAACAGAGC-3′, zo-1-Reverse 5′-GAAGAACAACCCTTTCATAAGC-3′, (198 bp amplicon); occludin-Forward 5′-CTTTGGCTACGGAGGTGGCTAT-3′occludin-Reverse 5′-CTTTGGCTGCTCTTGGGTCTG-3′, (86 bp amplicon); Claudin-1-Forward 5′-GCTGGGT-TTCATCCTGGCTTCT-3′, Claudin-1-Reverse 5′-CCTGAGCGGTCACGATGTTGTC-3′, (110 bp amplicon); GAPDH-Forward 5′-GAGACCTTCAACACCCCAGC-3′, GAPDH-Reverse 5′-ATGTCACGCACGATTTCCC-3′, (263 bp amplicon); IFN-γ-Forward 5′-ATGAACGCTACACACTGCATCTT-3′IFN-γ-Reverse 5′-TTTCTTCCACATCTATGCCACTT3′ (139 bp amplicon); TNF-α-Forward5′-GGTTCTGTCCCTTTCACTCACT-3′TNF-α-Reverse5′-GAGAAGAGGCTGAGACATAGGC-3′ (169 bp amplicon). Reaction system: 10 μL 2.5 × Real master Mix, 1.25 μL 20 × SYBR solution, 0.5 μL upstream primer, 0.5 μL downstream primer and 2 μL DNA template was brought up to 25 μL with purified water. The amplification was performed with Quantitect™ SYBR® Green PCR Mastermix (Qiagen), using the following time and temperature profile: 95°C for 5 min, 45 cycles of 1 min at 95°C, 10 s at 60°C. Fluorescent quantitative analysis was performed with the thermal cycler’s software package to calculate the ^△Ct^ value. The levels of zo-1, occludin, claudin-1, TNF-α and IFN-γ were calculated by the 2^-△△Ct^ analysis. The 2^-△△Ct^ was presented as the relative expression of the gene expression.

### Western blot analysis

The concentration of proteins of colon tissue and caco-2 cells were determined using coomassie brilliant blue assay, the extracts containing equal quantities of proteins (100 μg) were electrophoresed in 8% polyacrylamide gel. Subsequently, the separated proteins were transferred onto a nitrocellulose membrane. The membrane was blocked for non-specific binding for 30 min (5% skimmed milk in PBS), and then incubated overnight at 4°C with rabbit anti-zo-1 polyclonal antibody (1:100), mouse anti-claudin-1 monoclonal antibody (1:200), anti-occludin antibody (1:200), rabbit polyclonal anti-IFN-γ antibody (1:200), mouse monoclonal anti-TNF-α antibody (1:200) and rabbit anti-GAPDH monoclonal antibody (1:100). The membrane was subsequently incubated at room temperature for 2 h with goat anti-rabbit IgG (1:2 000)/anti-mouse IgG (1:2 000). Blots were developed with enhanced chemiluminescence detection reagents (Santa Cruz Biotechnology Inc), exposed on Kodak Xdmat blue XB-1 film and quantified by Bandscan 5.0 software using GAPDH as internal control. Densitometry is reported using the integral optical density value (IOD). The results were represented in the form of IOD ratio of the target protein to GAPDH.

### Statistical analysis

Data were expressed as mean ± standard deviation (mean ± SD) and analyzed with SPSS 13.0 software. The comparison of mean variability among all the groups was conducted by one-way ANOVA analysis and two group comparison with LSD test. A Student’s *t*-test was carried out for independent samples. Statistical significance was considered at *P* < 0.05.

## Results

### 1,25(OH)_2_D_3_ ameliorated clinical symptoms of established acute colitis model

**Figure 1:**
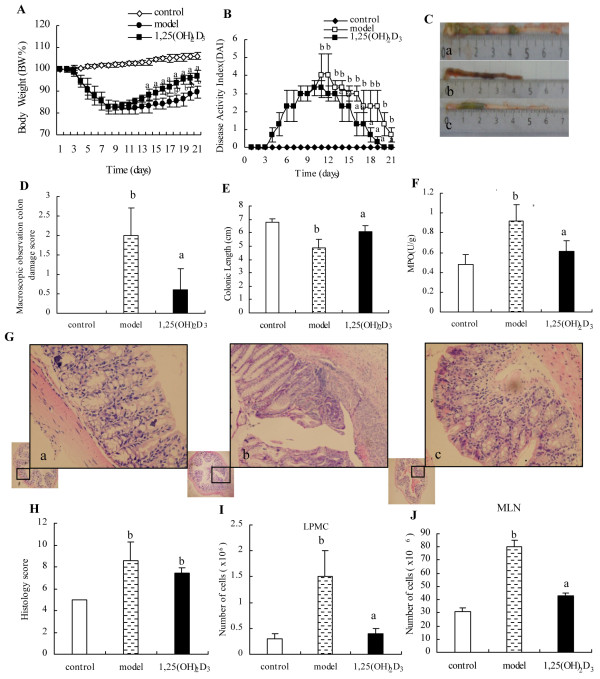
**The inflammation in the DSS-induced model of acute colitis.** (**A**) BW, shown as percentage of weight change, was assessed daily and represented from day 0 to day 21. Compared to the control group, the model group displayed a more weight loss of BW. (**B**) DAI, comprised of weight loss, stool consistency and OB, was shown. (**C**) Representative photographs of colon at the end of experiment were shown for control group (**a**), model group (**b**), and 1,25(OH)_2_D_3_-treated group (**c**). (**D**) Macroscopic inflammation was assessed and shown. (**E**) The length of the colon was quantitated and shown. (**F**) MPO enzymatic activity was measured and shown. (**G**) Representative H&E stained histology from control group (**a**), model group (**b**) and 1,25(OH)_2_D_3_-treated group (c) (H&E staining; original magnifications, ×200). (**H**) Colon histological scores in control, model and 1,25(OH)_2_D_3_-treated group were shown. (**I** and **J**) The total number of mononuclear cells from MLNs and LP was more in the model group compared with that in the control group. Data were expressed as mean ± SD. ^a^*P* < 0.01 *vs* model group; ^b^*P* < 0.01 *vs* control group.

As expected, the serum calcium concentrations in model group (1.74 ± 0.07) mmol/L was lower than that in control group (2.35 mmol/L ± 0.06 mmol/L, *P* < 0.01) and in 1,25(OH)_2_D_3_-treated group (2.60 mmol/L ± 0.07 mmol/L, *P* < 0.01). DSS caused the damage of the colon mucosal barrier, leading to gut inflammation and weight loss. We observed that mice in the model group had significantly greater weight loss than that in the control group from day 3 to day 8. Mice in 1,25(OH)_2_D_3_-treated group had similar degree of weight loss as mice in the model group from day 3 to day 8. However, mice in the 1,25(OH)_2_D_3_-treated group had rapid weight recovery from day 9 to day 21 than those in the model group (Figure1A) and the AUC of BW in the model group was smaller than that in the control group (1841.75 ± 52.11 *vs* 2195.60 ± 21.26, *P* < 0.01) and in the 1,25(OH)_2_D_3_-treated group (1841.75 ± 52.11 *vs* 1902.64 ± 31.61, *P* < 0.01). Similarly, mice in the 1,25(OH)_2_D_3_-treated group had firmer stool and less blood in the stool than those in the model group from day 9 to day 21. DAI, consisting of weight loss, stool consistency and OB, were measured daily, and the percentage of weight change of model group was shown in Figure1B and the AUC of DAI in model group was larger than that in the control group (43.78 ± 8.95 *vs* 0.00 ± 0.00, *P* < 0.01) and in the 1,25(OH)_2_D_3_-treated group (43.78 ± 8.95 *vs* 32.52 ± 6.49, *P* < 0.01).

### 1,25(OH)_2_D_3_ inhibited colonic inflammation

Mice in the model group had significantly worse macroscopic inflammation than those in the 1,25(OH)_2_D_3_-treated group (Figure1C and D). Colon shortening was used as a marker of inflammation. Consistent with the macroscopic inflammatory score, we found that by day 8, the colon length, in the model group was shorter than that in the control group (4.88 ± 0.63 cm *vs* 6.80 ± 0.27 cm, *P* < 0.01) and in the 1,25(OH)_2_D_3_-treated group (4.88 ± 0.63 cm *vs* 6.10 ± 0.42 cm, *P* < 0.01) (Figure1E). In addition, mice in the model group had significantly higher MPO activity than those in the 1,25(OH)_2_D_3_-treated group (0.92 ± 0.16 *vs* 0.61 ± 0.11, *P* < 0.01) and the control group (0.92 ± 0.16 *vs* 0.48 ± 0.10, *P* < 0.01) (Figure1F).

**Figure 2:**
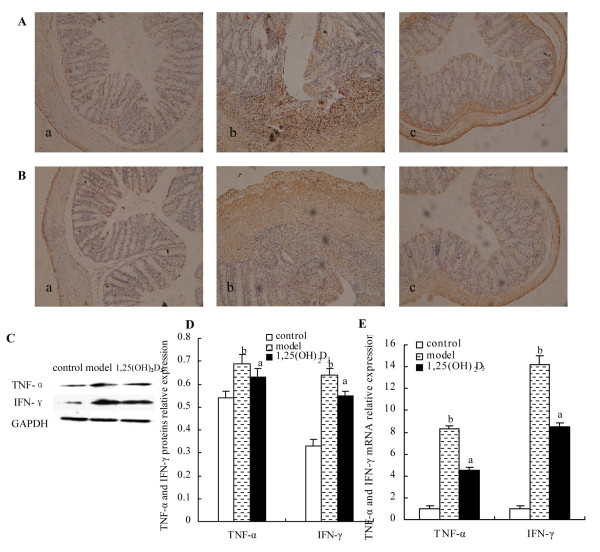
**Effect of 1,25(OH)**_**2**_**D**_**3**_**on the expressions of TNF-α and IFN-γ.** (**A** and **B**) Expressions of TNF-α and IFN-γ proteins were analyzed by immunohistochemistry in colon tissue from control group (**a**), model group (**b**) and 1,25(OH)_2_D_3_-treated group (**c**) (Immunohistochemistry staining; original magnifications, ×200). (**C** and **D**) Expressions of TNF-α and IFN-γ proteins in colon tissue were analyzed by Western blot. (**E**) Expressions of TNF-α mRNA and IFN-γ mRNA in colon tissue were analyzed by Real time Q-PCR. Data were expressed as mean ± SD. ^a^*P* < 0.01 *vs* model group; ^b^*P* < 0.01 *vs* control group.

Compared to the control group, histological examination of the colon in the model group showed extensive ulceration of the epithelial layer, edema, crypt damage of bowel wall, fibrosis of the muscularis mucosae, infiltration of granulocytes and mononuclear cells into the mucosa. Compared to mice in the model group, the mice treated with 1,25(OH)_2_D_3_ had a reduced DSS-induced histologic colitis (Figure1G). Histologic score includes: severity of inflammation, extent of injury, crypt damage, each score was multiplied by a factor equivalent with the percentage of tissue involvement, then the histology score is the sum of all parts of score. Our data showed that histology score in the model group had significantly higher than that in the control group (8.60 ± 2.20 vs 5.00 ± 0.00, *P* < 0.01), however, histology score in the 1,25(OH)2D3-treated group had no significantly lower than that in the model group (7.40 ± 0.80 *vs* 8.60 ± 2.20, *P* > 0.05) (Figure1H). The number of mononuclear cells from the MLNs and LP was more in the model group than that in the control group (MLNs: 80.33 × 10^6^ ± 14.51 × 10^6^*vs* 30.67 × 10^6^ ± 3.06 × 10^6^, *P* < 0.01; LPMC: 1.5 × 10^6^ ± 0.5 × 10^6^*vs* 0.3 × 10^6^ ± 0.1 × 10^6^, *P* < 0.01). Compared to mice of the model group, mice treatment with 1,25(OH)_2_D_3_reduced the number of mononuclear cells from the MLNs and LP (MLNs: 42.67 × 10^6^ ± 2.52 × 10^6^; LP: 0.4 × 10^6^ ± 0.1 × 10^6^, *P* < 0.01) (Figure1I and J). To determine the anti-inflammatory effect of 1,25(OH)_2_D_3_ on the DSS-induced colitis, inflammatory markers were used for immunohistochemistry including TNF-α and IFN-γ on colon tissues in the control group(Figure2A a and Figure2B a), the expressions of the TNF-α and IFN-γ were not seen and there were significantly increased expressions of the TNF-α and IFN-γ in the model group(Figure2A b and Figure2B b) than those in the 1,25(OH)_2_D_3_-treated group(Figure2A c and Figure2A c). The protein levels of these inflammatory cytokines in the model group were higher than those in the control group by Western blot (TNF-α: 0.69 ± 0.04 *vs* 0.54 ± 0.03, *P* < 0.01; IFN-γ: 0.64 ± 0.03 *vs* 0.33 ± 0.03, *P* < 0.01) and in the 1,25(OH)_2_D_3_-treated group (TNF-α: 0.69 ± 0.04 *vs* 0.63 ± 0.04, *P* < 0.01; IFN-γ: 0.64 ± 0.03 *vs* 0.55 ± 0.02, *P* < 0.01) (Figure2C and D). Mice treated with 1,25(OH)_2_D_3_ had reduced mRNA levels of inflammatory cytokines when compared to those in the model group (TNF-α: 4.48 ± 0.08 *vs* 8.34 ± 0.11, *P* < 0.01; IFN-γ: 8.5 ± 0.34 *vs* 14.18 ± 0.86, *P* < 0.01) (Figure2E). Together, these data indicated that 1,25(OH)_2_D_3_ may ameliorate the inflammation in the colitis model.

### 1,25(OH)_2_D_3_ attenuated the DSS-induced paracellular permeability

**Figure 3:**
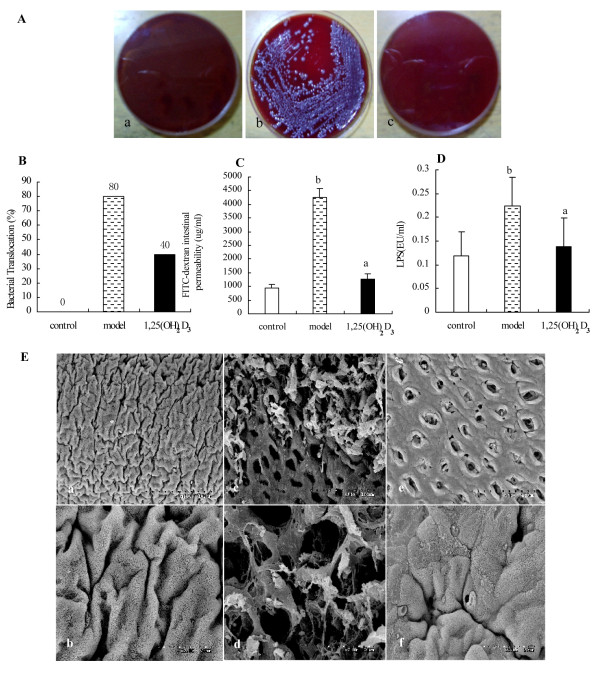
**1,25(OH)**_**2**_**D**_**3**_**attenuated the loss of intestinal barrier during DSS-induced colitis model.** (**A**) Detection of viable bacteria in MLNs were shown for control group (**a**), model group (**b**) and 1,25(OH)_2_D_3_-treated group (**c**). (**B**) Differences in incidence of bacterial translocation were quantitated and shown, (**C**) Quantification of serum FITC-D, a measure of intestinal barrier function, was shown. (**D**) The levels of endotoxin were compared between control group, model group and 1,25(OH)_2_D_3_-treated group. Data were expressed as mean ± SD. ^a^*P* < 0.01 *vs* model group; ^b^*P* < 0.01 *vs* control group. (**E**) The ultrastructural features were observed by SEM. The colon mucosa in the control group was regular without histological lesion, (**a**, ×500; **b**, ×2 000); In the model group, the mucosa was severe loss, with histological lesions including crypt distortion and abscesses, (**c**, ×500; **d**, ×2 000); Areas with histological lesions in the 1,25(OH)_2_D_3_-treated group were significantly ameliorated; However, compared with the control group, there were less lesions and the crypt distortion and abscesses were less severer. Futhermore, crypt openings were disposed in rows. (**e**, ×500; **f**, ×2 000).

To investigate the effect of 1,25(OH)_2_D_3_ on paracellular permeability, we examined intestinal permeability in animals subject. One of measures of permeability was bacterial translocation. Our study showed 1,25(OH)_2_D_3_ significantly reduced bacteria translocation to the MLNs in the 1,25(OH)_2_D_3_-treated group when compared to the model group (Figure3A and B). Another measure of intestinal permeability was FITC-D permeability. Our data showed that the mice in the 1,25(OH)_2_D_3_-treated group had a lower intestinal permeability to 4-kDa FITC-D when compared to that in the model group (Figure3C). Consistent with reduced bacterial translocation, there was a result that the blood LPS levels in the 1,25(OH)_2_D_3_-treated group was lower than that in the model group (Figure3D). These results suggested that 1,25(OH)_2_D_3_ may maintain intestinal epithelial integrity by decreasing paracellular permeability.

### 1,25(OH)_2_D_3_ ameliorated DSS induced ultrastructural changes

SEM was used to observe ultrastructural features of the colon in different groups. We found that the colon in the model group had severer loss of the mucosa with typical histological inflammation, including crypt distortion compared to that in the control group (Figure3E a, b). Additionally, a greater number of enterocytes from the mice in the model group (Figure3E c, d) showed less of the glycocalyx, and more irregular of the surface than that in the control group. Meanwhile, mice treatment with 1,25(OH)_2_D_3_ showed ameliorated ultrastructural changes (Figure3E e, f). These data suggest that 1,25(OH)_2_D_3_ may reduce intestinal permeability in part by preserving ultrastructural integrity of the epithelial mucosa.

### 1,25(OH)_2_D_3_ prevented DSS-induced disruption of TJ

**Figure 4:**
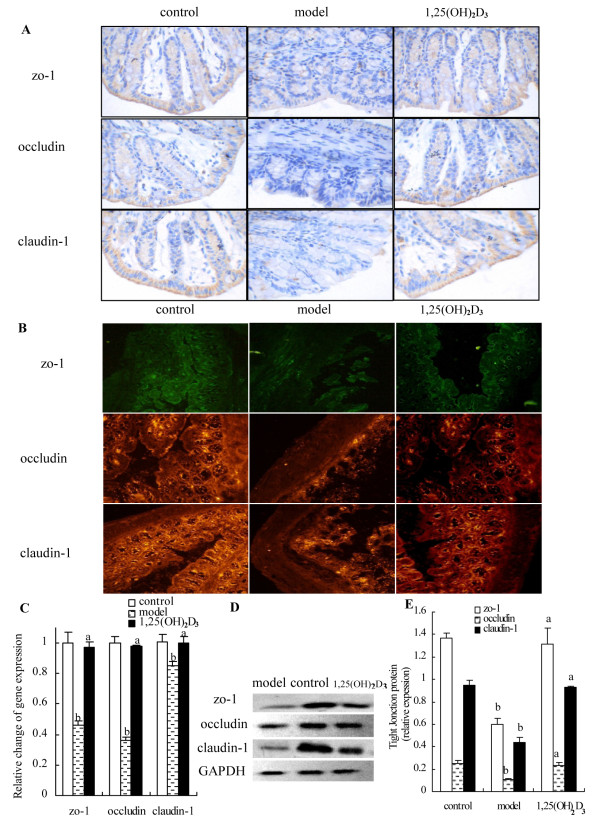
**Effect of 1,25(OH)**_**2**_**D**_**3**_**on colonic epithelial junctions.** (**A**) Expressions of zo-1, occludin and claudin-1 were examined by immunostaining for control, model, and 1,25(OH)_2_D_3_-treated group. Magnification: ×200. (**B**) Immunoflorescent stain with anti-claudin-1 (red), anti-oocludin (red) and anti-zo-1 (green) antibody was shown. Notes of the zo-1, occludin and claudin-1 staining were markedly decreased in the model group compared with that of the control and 1,25(OH)2D3-treated group. (**C**) Expressions of zo-1, occludin and clandin-1 genes were performed and shown by real time Q-PCR analysis. (**D** and **E**) Expressions of TJ proteins in the colon were assessed by Western blot. In model group, expressions of zo-1, occludin and claudin-1 were decreased markedly compared with that of control group. Data were expressed as mean ± SD. ^a^*P* < 0.01 *vs* model group; ^b^*P* < 0.01 *vs* control group.

TJ complexes interact with the actin cytoskeleton at the apical end of the lateral membranes. To determine the protective effect of 1,25(OH)_2_D_3_ on the DSS-induced disruption of TJ, TJ markers were used for immunohistochemistry including zo-1, occludin and claudin-1 on colon epithelium. In the control group, TJ proteins were seen on the cellular membrane of the epithelial cells, mostly in the spinous and granular layers. In the 1,25(OH)_2_D_3_-treated group, the expression of the TJ increased in both the cellular membrane and cytoplasm of spinous and granular layers in the mucosa. However, in the model group, expressions of the TJ decreased in individual cells of the spinous and granular layers (Figure4A and B). To determine whether 1,25(OH)_2_D_3_ could prevent DSS-induced reduction in TJ expression, we performed real time Q-PCR analysis for TJ markers message levels (Figure4C). We demonstrated that when compared to TJ expressions in control group, in the model group there were a significantly reduced expressions in zo-1, occludin and clandin-1 (0.46 ± 0.03 *vs* 1 ± 0.07, 0.36 ± 0.02 *vs* 1.00 ± 0.04, 0.85 ± 0.05 *vs* 1.00 ± 0.05, respectively; *P* < 0.01). We also found that TJ marker expressions in the 1,25(OH)_2_D_3_-treated group were also significantly higher than that in the model group (zo-1: 0.97 ± 0.04, occludin: 0.98 ± 0.01, clandin-1: 1.00 ± 0.04; *P* < 0.01) (Figure4D and E), which suggested the importance of 1,25(OH)_2_D_3_ to maintain the integrity of the junction complex.

### The effect of 1,25(OH)_2_D_3_ on the structure and function of the junction complexes *in vitro*

**Figure 5:**
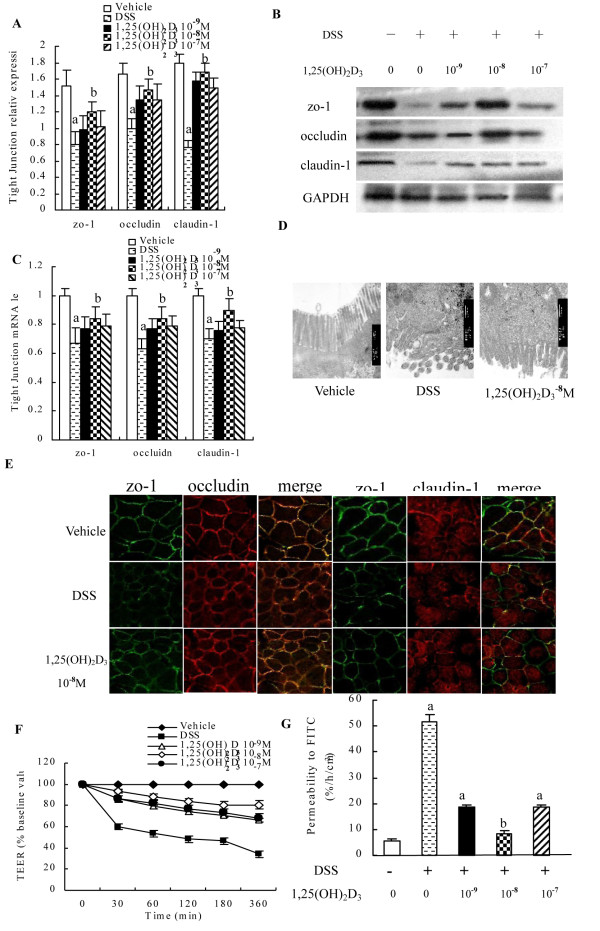
**Effect of 1,25(OH)**_**2**_**D**_**3**_**on the barrier function*in vitro.*** (**A** and **B**) Induction of TJ proteins by 1,25(OH)_2_D_3_ in Caco-2 cells. Caco-2 cells were treated with indicated dose of 1,25(OH)_2_D_3_ for 48 h, and cell lysates were analyzed by Western blot. Western blot analysis showed induction of zo-1, occludin and claudin-1 proteins after 48 h of adding to1,25(OH)_2_D_3_. (**C**) Expressions of zo-1, occludin, claudin-1 mRNA were assessed after adding to 1,25(OH)_2_D_3_ by real time Q-PCR. (**D**) TEM showed the disruption of the TJ, desmosomes, microvillus and brush border on the apical side of Caco-2 cell monolayer in the model group compared to that of the vehicle and 1,25(OH)2D3-treated group. (**E**) Cell monolayer was stained for occludin (red), claudin-1 (red) and zo-1(green) by immunofluorescence staining, noting the disruption of the TJ and reduction of fluorescence intensity in DSS group. (**F**) Effect of 1,25(OH)_2_D_3_ on TEER. A dose-dependent recovery of TEER with 1,25(OH)_2_D_3_-treated was seen. (**G**) Paracellular FITC-D was measured with or without 2% DSS in the absence or presence of 1,25(OH)_2_D_3_ at 10^−9^, 10^−8^, 10^−7^ M (administered 2 h prior to DSS) for 48 h, 1,25(OH)_2_D_3_ attenuated the epithelial hyperpermeability induced by DSS. Data were expressed as mean ± SD. ^a^*P* < 0.05 *vs* vehicle group, ^b^*P* < 0.05 *vs* DSS group.

To further address molecular mechanisms of the protective role of 1,25(OH)_2_D_3_ in intestinal barrier function, we used an established *in vitro* Caco-2 cells culture system. Caco-2 cells formed TJ when grown to monolayer on membrane filters. As in the case of Caco-2 cells, the addition of 1,25(OH)_2_D_3_ to the Caco-2 cells for 48 h increased levels of TJ proteins and mRNA such as zo-1, claudin-1, and occludin, and the optimum dose occurred at 10^-8^ M (Figure5A, B and C). Further examination of these Caco-2 cell monolayer with TEM revealed intact TJ, desmosomes, microvillus and brush border on the apical side of Caco-2 cell monolayer in the 1,25(OH)_2_D_3_-treated group (Figure5D). Consistently, immunostaining with anti-zo-1, claudin-1, and occludin antibodies showed that 1,25(OH)_2_D_3_ (10^-8^ M) markedly enhanced TJ expression in the Caco-2 monolayer, and reflected by bright staining in the membranes of the treated cells. When the cells were incubated with 2% DSS for 2 h, the TJ on the monolayer was markedly disrupted. In contrast, in the presence of 1,25(OH)_2_D_3_, the TJ was resistant to the DSS damage and remained intact, as evidenced by a strong staining of the zo-1, claudin-1, and occludin on the membranes between the cells (Figure5E). When Caco-2 monolayer was incubated with 2% DSS, the TEER gradually decreased over 6 h in the DSS group. However, in the 1,25(OH)_2_D_3_-treated cells, the TEER was moderately reduced over the following 6 h (Figure5F). Interestingly, FITC-D permeability results showed that DSS increased the FITC-D permeability in the DSS group, compared with that of the vehical group (51.83 ± 2.48 *vs* 5.5 ± 1.05, *P* < 0.01), while the administration of 1,25(OH)_2_D_3_ (10^-9^ M, 10^-8^ M, 10^-7^ M) afforded cytoprotection against DSS-induced monolayer hyper-permeability of Caco-2 cell monolayer (18.5 ± 1.05 *vs* 51.83 ± 2.48, 8.17 ± 1.17 *vs* 51.83 ± 2.48, 18.5 ± 1.05 *vs* 51.83 ± 2.48, *P* < 0.01) in the 1,25(OH)_2_D_3_-treated cells. Our data showed that 1,25(OH)_2_D_3_(10^-8^ M) had a lower monolayer permeability to FITC-D when compared to that 1,25(OH)_2_D_3_(10^-9^ M, 10^-7^ M). Therefore, the optimal concentration of 1,25(OH)_2_D_3_ on Caco-2 is 10^-8^ M for FITC-D permeability (Figure5G). These data suggest that 1,25(OH)_2_D_3_ might help to maintain epithelial barrier integrity by preserving the expression of junction proteins and increase its resistance to DSS damage. These *in vitro* results are consistent with the observation *in vivo* that 1,25(OH)_2_D_3_ reduces DSS induced colitis in part by preserving TJ integrity of the epithelial mucosa.

## Discussion

The intestinal mucosal integrity is a physical and metabolic barrier against toxins and pathogens in the lumen. The barrier regulates macromolecule trafficking between the lumen and the internal milieu and protects the host by preventing harmful solutes, microorganisms, toxins, and luminal antigens from entering the body [36]. Compromise in intestinal barrier function can result in an increased exposure of the host to luminal antigens and pathogens, leading to inflammation [37]. The epithelial barrier function is largely determined by intercellular TJ. The TJ are responsible for restricting paracellular movement of compounds across the intestinal mucosa [38,39].

1,25(OH)_2_D_3_ was found to protect the intestinal mucosa from various insults [40]. For example, 1,25(OH)_2_D_3_ may enhance defense mechanisms in the gastrointestinal mucosa and prevent mucosal injury in IBD [16]. In the present study, administration of 1,25(OH)_2_D_3_ was shown to ameliorate signs and symptoms of inflammation, such as colon shortening, weight loss, and increased DAI. Intestinal mucosa has crucial functions in regulating intestinal homeostasis by strictly protecting the subepithelial compartment from potentially noxious luminal compounds [41,42]. Kong et al. [6] have proved that vitamin D deficiency may compromise the mucosal barrier, leading to increased susceptibility to mucosal damage and increased risk of IBD. Intestinal barrier dysfunction is one of the major contributing factors in multiple pathological conditions of the gastrointestinal tract. An increased permeability in the TJ may provide a major site for both infection and establishment of inflammation in the gut [43-45]. Bacterial translocation is believed to occur via a paracellular pathway through the epithelial cells. Our data showed 1,25(OH)_2_D_3_ might have a protective effect on barrier integrity by maintaining the expression of TJ proteins, thereby reducing the severity of gut inflammation. This study adds to previous reports suggest that bioavailability of 1,25(OH)_2_D_3_ is an important contributing factor for determining the epithelial integrity.

Once the mucosal barrier is breached, the submucosa is exposed to a vast pool of luminal antigens, including food and bacteria, thereby engaging the innate immune responses including increased production in pro-inflammatory cytokines TNF-α and IFN-γ. We showed that the addition of 1,25(OH)_2_D_3_ was shown to ameliorate the DSS-induced high-expression in TNF-α and IFN-γ. The reduction in pro-inflammatory cytokines by 1,25(OH)_2_D_3_ may be either due to its direct suppressive effect on the expression of these pro-inflammatory cytokines or due to the effect on maintenance of epithelial barrier function, leading to a reduction in foreign luminal antigenic load, and a full activation of the innate immune system.

## Conclusion

In summary, our data suggest that 1,25(OH)_2_D_3_ may be effective in ameliorating DSS-induced acute colitis, gauged by reducing clinical symptom, decreasing macroscopic and histological inflammation, enhancing epithelial cell resistance to injury and suppressing pro-inflammation responses to luminal antigens. Although some parts of the histology score are not obviously ameliorated by 1,25(OH)_2_D_3_, we think that there are some reasons, such as: a short treatment time, a very slow process of pathological change and so on. However, the effect of 1,25(OH)_2_D_3_ in IBD is worth further study. Our data showed that 1,25(OH)_2_D_3_ may play an important role in maintaining epithelial integrity and regulating the innate immune responses. The therapeutic potential of 1,25(OH)_2_D_3_ for the treatment of human colitis warrants exploration.

## Competing interests

The authors declare that they have no competing interests. This work was supported by public health bureau grant of Hebei province (No. 20090104) to XL Zhang. Non-financial competing interests. Non- patents competing interests. Non-any stocks or shares in an organization.

## Authors’ contributions

HZ, HZ and HW participated in the design of the study and performed the statistical analysis and drafted the manuscript. HL and LL conceived of the study; JG and CL collected of data and analyzed histological scores; XZ and DQS participated in its design and coordination, helped to draft the manuscript and have given final approval of the version to be published. All authors read and approved the final manuscript.
